# The Ratio of Body Weight/Length Squared Relates to Low Serum α-Tocopherol in Preterm Infants

**DOI:** 10.7759/cureus.76575

**Published:** 2024-12-29

**Authors:** Paraskevi Detopoulou, Panos Papandreou, Maria Skouroliakou

**Affiliations:** 1 Department of Nutritional Sciences and Dietetics, University of Peloponnese, Kalamata, GRC; 2 Department of Nutrition, IASO Hospital, Athens, GRC; 3 Department of Nutrition and Dietetics, Harokopio University, Athens, GRC

**Keywords:** bmi, length, neonates, preterm, vitamin e, weight, α-tocopherol

## Abstract

Introduction: Preterm infants are at high risk of developing α-tocopherol deficiency, since fat depots are low, intake may be insufficient, malabsorption may coexist, and dietary needs are high. Data on predictors of low α-tocopherol are still limited. Thus, this study aimed to assess the levels of α-tocopherol in preterm infants at birth and explore its anthropometric predictors.

Methods: Preterm infants (n=84) from a neonatal intensive care unit were studied. Weight, length, head circumference, and gestational age were recorded. The measurement of α-tocopherol levels was performed in the first 24 hours with high-performance liquid chromatography (HPLC).

Results: Logistic regression models were applied to identify factors related to low α-tocopherol levels (<1.5 mg/L). The median gestational age was 29.5 weeks and the mean birth weight was 1254 g. Most neonates were of very low birth weight (~89%). About 31% of preterm infants had α-tocopherol deficiency (cutoff <1.5 mg/L). In logistic regression analysis, a newly proposed index, i.e., weight/length^2^ (measured in g/cm^2^), was related to low vitamin status. More particularly, a ratio >0.81 g/cm^2^ was related to α-tocopherol deficiency independently of other covariates.

Conclusion: Further studies are needed to prove the usefulness of this "easy-to-measure" proposed index in the early detection of α-tocopherol deficiency.

## Introduction

Vitamin E naturally exists in eight forms (four tocopherols and four tocotrienols). The form of α-tocopherol is the most common of these structures in humans [1]. Vitamin E has a strong antioxidant action [1] and is implicated in protection against inflammation and cancer [2], and during gestation, it is involved in embryo development and placenta maturation [1]. Serum levels of vitamin E may be affected by age, intake, and circulating lipids [3]. Preterm infants are at high risk of developing vitamin E deficiency as fat depots are low, ingestion may be insufficient, poor absorption may coexist, and dietary requirements are high [4]. Preterm neonates have been previously reported to have low vitamin E concentrations in some studies [5]. In a previous study of our group, it was shown that 10% of preterm neonates were deficient in retinol, while 20% of them were deficient in α-tocopherol [6]. In addition, in the same study, α-tocopherol was positively associated in a non-linear way with gestational age [6], indicating that preterm neonates may be at risk of α-tocopherol deficiency. Vitamin E deficiency in neonates is related to several infant pathologies, such as hemolytic anemia, intraventricular hemorrhage, bronchopulmonary dysplasia, ataxia, developmental delay, and others [7].

Oxidative stress is thought to be a central etiological mechanism for several of the aforementioned conditions. Indeed, preterm birth exposes the fetus to an oxidative environment, since oxygen is inhaled [8]. In addition, infant cell membranes are more prone to oxidation due to their high polyunsaturated fatty acid content [8]. Furthermore, oxygen resuscitation and common practices in the intensive care unit such as ventilation, parenteral nutrition, and blood transfusions increase the release of free radicals, which further increase oxidative stress [8]. In preterm neonates, the responses to oxidative burden and the antioxidant potential are suboptimal, making them more vulnerable to the consequence of oxidative stress [8]. Therefore, preterm neonates may have decreased levels of antioxidant enzymes, vitamins, and minerals, such as glutathione peroxidase, superoxide dismutase, catalase, vitamins A and E, selenium, copper, zinc, and others [9].

Although the American Academy of Pediatrics recommends using weight-for-length curves for children up to two years [10], several data suggest the increasing usefulness of the weight/length^2^ ratio or body mass index (BMI) in predicting adiposity [11]. On the contrary, lower values of weight/length^2^ at birth are associated with an increased risk of bronchopulmonary dysplasia, necrotizing enterocolitis, and death after taking into account the birth weight z-score [12]. This observation suggests that the disproportionality of weight and length in neonates may adversely affect the neonate's health [11]. In addition, maternal vitamin E has been positively associated with birth weight [13], while cord vitamin E has been negatively associated with birth weight [14].

Given the harmful effects of oxidative stress and α-tocopherol deficiency in preterm infants, it is important to evaluate α-tocopherol status early. In addition, it is important to identify correlates of low levels of α-tocopherol, in cases where it cannot be measured directly. Thus, the study aimed to evaluate the levels of α-tocopherol in preterm neonates at birth and explore its correlates, such as anthropometry-related ones. To our knowledge, no other studies have investigated the relation of BMI or related anthropometric indices to α-tocopherol in neonates.

## Materials and methods

Study design and participants

This was a cross-sectional study of 84 preterm infants from the neonatal intensive care unit (NICU) at IASO Hospital (Athens, Greece). This work was supplementary to a previous work of our group, which assessed retinol and α-tocopherol levels in a smaller sample of preterm neonates [6]. The Scientific Committee of IASO Hospital approved both protocols (approval number: A21112018). The work presented has been carried out following the Code of Ethics of the World Medical Association and the Declaration of Helsinki for humans.

This was a prospective study, and subjects were included on a "first come-first included" basis, provided they gave their consent. Preterm neonates admitted to the NICU of the maternity hospital were included (January 2021-June 2021). The inclusion criteria were (i) gestational age of 26-35 weeks and (ii) parental consent for inclusion in the study. The exclusion criteria were the following: (i) gestational diabetes or pre-eclampsia, (ii) congenital infections and anomalies, (iii) perinatal asphyxia, and (iv) absence of parental consent. It is noted that at the time of measurements, the neonates had not received corticosteroids after birth. Parents of the infants signed an informed consent form before the enrolment of infants in the study.

The participating mothers were of Greek nationality and resided in Athens or nearby suburbs of Attica. It is noted that all mothers were non-smokers.

Anthropometric and biochemical measurements

A digital scale was used to measure weight (0.005 kg accuracy, model A&D SK-WP, 1-10 kg (Toshima City, Tokyo, Japan)). The crown-heel length was measured with a neonatometer (Harpenden Neonatometer, Holtain, Crymych, UK) with a 1 mm precision. Head circumference was measured with a non-stretch tape to the nearest 0.1 cm. If birth weight was lower than 2500 g or lower than 1500 g, neonates were characterized as having low birth weight and very low birth weight, correspondingly [15]. The index weight/height^2^ was compared to previously proposed appropriate for gestational age and sex median values of preterm infants [16].

Approximately 3 ml of blood was collected the following day of the birth. After centrifugation (3000 rpm × 10 minutes), serum was collected and kept at -20°C. The measurement of α-tocopherol was performed with high-performance liquid chromatography (HPLC) (Agilent, 1100, constant-flow pump, Santa Clara, CA, USA) with an ultraviolet (UV) detector set at 295 nm and a commercially available reagent kit (Chromsystems, Gräfelfing, Germany). An isocratic elution program was used (solvent flow 1.5 ml/min, column temperature 25°C). Each sample was injected at a volume of 50 μl. The deficiency in α-tocopherol was defined at levels <5.0 mg/L, in line with the World Health Organization criteria [3], and severe deficiency was defined at levels <1.5 mg/L [17].

Statistical analysis

Normality was tested with the Kolmogorov-Smirnov criterion. Normally distributed variables are presented as means ± standard deviation, while skewed variables are presented as medians and interquartile range. Length was transformed into a squared variable to achieve normality.

For categorical variables, absolute numbers and frequencies (%) are shown. For comparisons between normally distributed and transformed continuous variables between the two groups, the t-test was applied. The chi-squared test was used for group comparisons (i.e., males and females). Spearman correlations were applied to identify potential non-linear relationships between variables or to identify correlations between non-parametric variables. Logistic regression models were constructed to identify variables that can predict low α-tocopherol status. The dependent variable was dichotomous, i.e., α-tocopherol <1.5 mg/L or >1.5 mg/L. As independent variables, various anthropometric indices and other covariates, such as sex and gestational age, were used. The significance level was set at 10%. The IBM SPSS Statistics for Windows, V. 23.0 (Released 2015, IBM Corp., Armonk, NY, USA), was used for analysis. In addition, scatter dot diagrams were performed with the Chart Builder of the SPSS.

## Results

The anthropometric characteristics and α-tocopherol status of preterm neonates are displayed in Table 1. Briefly, the median gestational age was 29.5 weeks (interquartile range 28.0-31.0 weeks), and the mean birth weight was 1254 g (standard deviation 255 g). Most neonates were of very low birth weight (~89%). The median head circumference was 28 cm, and its interquartile range was 27-29 cm. Concerning the ratio of birth weight/length^2^, it was 0.819 ± 0.129 g/cm^2^. Compared to published birth weight/length^2^ sex- and gestational age-specific curves of preterm infants [16], 83.5% of neonates were below the median value. More particularly, 84.8% of males and 86.5% of females had a ratio of weight/length^2^ below the median value for their gestational age (data not shown). The percentage of small-for-gestational-age children was 15.3%. About 31% of preterm infants had an α-tocopherol deficiency when the cutoff <1.5 mg/L was used, and 100% had an α-tocopherol deficiency when the cutoff <5.0 mg/L was used. No sex differences were detected.

**Table 1:** Anthropometric characteristics and vitamin status of preterm neonates. ^†^Values were transformed before statistical comparisons to achieve normality. *Low birth weight was defined as weight <2500 g. **Very low birth weight was defined as weight <1500 g. ^∫^α-Tocopherol deficiency was defined as α-tocopherol <1.5 mg/L. The significance level was set at 10%.

	Total sample	Males	Females	P-value
	(n=84)	(n=46)	(n=38)	
Gestational age (y)	29.5 (28.0-31.0)	30.0 (28.0-31.0)	29.0 (28.0-31.0)	0.657
Birth weight (g)	1254 ± 255	1268 ± 237	1236 ± 278	0.572
Low birth weight (%)*	9 (10.7)	5 (10.9)	4 (10.8)	0.638
Very low birth weight (%)**	75 (89.3)	41 (89.1)	34 (91.9)	0.485
Length (cm)^†^	39.0 (37.0-41.0)	39.5 (37.0-42.0)	39.0 (36.0-40.5)	0.392
Birth weight/length^2^ (g/cm^2^)	0.819 ± 0.129	0.815 ± 0.125	0.824 ± 0.136	0.761
Small-for-gestational-age (n, %)	13 (15.3)	9 (19.6)	4 (10.8)	0.275
Head circumference (cm)	28 (27-29)	28 (27-29)	27 (27-29)	0.322
α-Tocopherol (mg/L)	1.97 ± 0.76	1.93 ± 0.80	2.01 ± 0.73	0.641
α-Tocopherol deficiency (n, %)^∫^	26 (31)	46 (55.4)	37 (44.6)	0.516

Table 2 shows the Spearman correlations between α-tocopherol and several anthropometric variables. A negative association between α-tocopherol and weight/length^2^ was detected (p = 0.06), while no associations were identified between α-tocopherol and gestational age, head circumference, birth weight, or length.

**Table 2:** Spearman correlations between α-tocopherol and anthropometric variables in the total sample. Spearman correlation coefficients are displayed. The significance level was set at 10%.

	α-Tocopherol (mg/L)
Gestational age (y)	rho = 0.095
	p = 0.392
Birth weight (g)	rho = 0.018
	p = 0.875
Length (cm)	rho = 0.178
	p = 0.121
Head circumference (cm)	rho = -0.025
	p = 0.828
Birth weight/length^2^ (g/cm^2^)	rho = -0.212
	p = 0.064

In Table 3, the models of logistic regression are presented (crude and multi-adjusted models). As can be seen, weight/length^2^ >0.81 g/cm^2^ was positively related to low α-tocopherol status. Interestingly, the weight/length^2^ predicted low α-tocopherol levels independently of other covariates, i.e., gestational age, gender, and head circumference. It is noted that additional logistic regression models were applied with weight and length separately inserted in the model. However, weight and length alone were not able to predict low α-tocopherol levels (Exp(B): 1.001; 95% CI: 0.998-1.004; p = 0.548 for weight and Exp(B): 0.898; 95% CI: 0.718-1.122; p = 0.343 for length (data not shown)).

**Table 3:** Logistic regression models with vitamin E deficiency as a dependent dichotomous variable (deficiency = 1 vs no deficiency = 0). The significance level was set at 10%. *Reference category. CI: confidence interval; Exp(B): exponentiation of the B coefficient (odds ratio); NA: not applicable

	Crude model					Model 1					Model 2				
			95% CI					95% CI					95% CI		
	B	Exp(B)	Lower	Upper	P	B	Exp(B)	Lower	Upper	P	B	Exp(B)	Lower	Upper	P
Gestational age (weeks)	-0.255	0.775	0.568	1.057	0.108	-0.261	0.770	0.563	1.055	0.104	-0.283	0.754	0.543	1.046	0.090
Gender (males vs. females*)	-	-	-	-	-	0.179	1.196	0.411	3.484	0.743	0.127	1.136	0.387	3.336	0.817
Head circumference (cm)	-	-	-	-	-	-	-	-	-	-	0.095	1.099	0.771	1.566	0.601
Weight/length^2^ (g/cm^2^) (≤0.81 g/cm^2^*vs >0.81 g/cm^2^)	1.684	5.389	1.575	18.446	0.007	1.714	5.551	1.588	19.406	0.007	1.542	4.675	1.330	16.431	0.016

**Figure 1:**
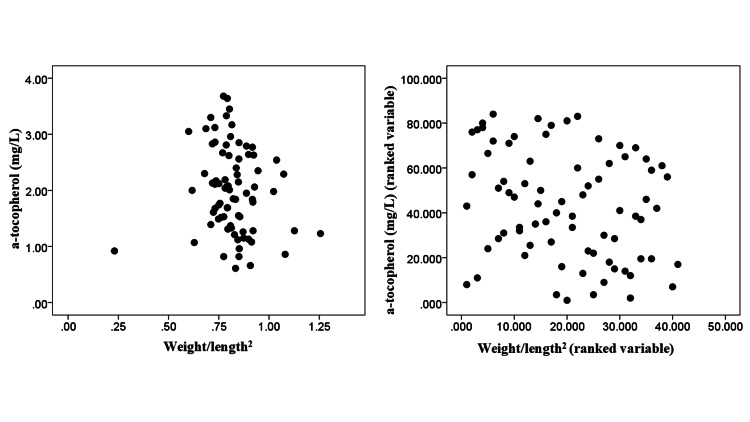
Scatter dot diagram between α-tocopherol and weight/length squared (raw and ranked variables).

The relation of the index weight/length^2^ to α-tocopherol levels is also graphically presented in Figure 1. It is noted that graphs with both raw values and ranked values of weight/length^2^ and α-tocopherol are shown in Figure 1.

## Discussion

The present study assessed α-tocopherol levels in preterm infants and aimed to identify anthropometric correlates of α-tocopherol deficiency. About one-third of preterm infants had α-tocopherol deficiency (cutoff <1.5 mg/L). In multi-adjusted logistic regression analysis, a ratio of weight/length^2^ >0.81 g/cm^2^ related to low vitamin status.

Few studies have evaluated vitamin E deficiency by defining different cutoffs from 1.5 mg/L to 5.0 mg/L [18]. In the present study, about 31% of preterm infants had α-tocopherol deficiency when the cutoff <1.5 mg/L was used, and 100% had α-tocopherol deficiency when the cutoff <5.0 mg/L was used. As recently reviewed, vitamin E deficiency in neonates has a high variability ranging from 19% to 100% [18]. Levels of α-tocopherol in Greece have been found at 2.04 ± 0.63 to 2.47 ± 0.78 mg/L in an intervention study in preterm infants with a smaller sample [19] and 6.83 ± 3.02 mg/L in a recent study of 30 preterm infants [6]. Our results (mean 1.97 ± 0.76 mg/L) are comparable with the study of Skouroliakou et al. [19] but not fully comparable with those of Papandreou et al. [6], although a similar detection methodology with the latter was followed. In addition, Papandreou et al. showed that 20% of preterm neonates were deficient in α-tocopherol when the cutoff <5.0 mg/L was used [6], while in the present study, all neonates were deficient with this criterion. In parallel, there is no overall consensus regarding optimal α-tocopherol levels in preterm neonates. The European Society for Paediatric Gastroenterology Hepatology and Nutrition (ESPGHAN), the European Society for Clinical Nutrition and Metabolism (ESPEN), the European Society for Paediatric Research (ESPR), and the Chinese Society of Parenteral and Enteral Nutrition (CSPEN) jointly recommend treatment in α-tocopherol levels below 1-2 mg/L [20].

The ratio of weight/length^2^ was inversely related to α-tocopherol deficiency in multi-adjusted logistic regression models. It is noted that most infants in the present study were below the recently reported median values for weight/length^2^ curves for preterm infants [16]. Other researchers have reported a positive relation between α-tocopherol and birth weight (without including preterm born infants) [21] or gestational age [17]. In several studies, vitamin E status was comparable between preterm and term infants [5]. In parallel, adiposity may affect nutritional status. For example, obese children may store lipid-soluble vitamins in adipose tissue, and thus, concentrations of fat-soluble antioxidants may be low [22].

It is most probable that the association found in this study reflects the fact that fat-soluble vitamins are stored in larger quantities as fat depots increase. However, weight may not adequately reflect adipose tissue mass, since it also includes skeletal mass. The ratio of weight/length^2^ considers the contribution of skeletal mass, which constitutes a larger part of total birth weight in preterm than in term infants (since fat depots are smaller). A study including ~1000 neonates measured body composition with the use of air displacement plethysmography across different ratios of weight/length^2^ [23]. It was documented that neonates with a low weight/length^2^ ratio (less than the third percentile) had less than half body fat percentage and fat mass/fat-free mass ratio than neonates with a normal weight/length^2^ ratio [23]. It is thus shown that the weight/length^2^ ratio in low-birth-weight neonates denotes a larger decrease in body fat compared to fat-free mass, which may explain the association of the weight/length^2^ ratio with lipid-soluble vitamins.

The investigated index has not been largely used in neonates and infants. In a US sample of 391,681 infants, the ratio of weight/length^2^ was the best predictor of body proportionality in preterm neonates [24]. In addition, it has been proposed that weight-for-length or other indices of body proportionality should be routinely used for the growth assessment of infants in the NICU [25]. Furthermore, the ratio of weight/length^2^ in preterm neonates has been correlated with dual-energy X-ray absorptiometry-derived body composition variables, such as fat mass percentage, fat mass, fat-free mass, and bone mineral content [26]. Increases in weight/length^2^ z score in neonates born <30 weeks have been related to a higher probability of bronchopulmonary dysplasia or death [27]. Other studies have shown that weight/length^2^ is a better indicator of adiposity than the ratio of weight to length at 1-5 months of age [28].

The strengths of the study include that an appropriate methodology (HPLC) was used for the analysis of tocopherols. In this study, corticosteroids had not been administered to neonates at the time of measurements. This is important, since dexamethasone may interfere with circulating α-tocopherol and anthropometric measurements. Indeed, dexamethasone has been connected to alterations in α-tocopherol transfer protein in rats [29]. It is noted, however, that maternal steroids were administered before 34 weeks. In addition, the present work proposes an easy-to-measure index, which is related to low α-tocopherol levels.

Some limitations of the present work should be stated. Firstly, all women had caesarian delivery, which may affect fat-soluble vitamin levels [30]. Moreover, the present study did not include a control group of full-term infants to capture potential differentiated associations of anthropometric indices and vitamin E in term infants. There were no accessible data on the nutritional management of infants. However, the samples were taken 24 hours after birth, so it is unlikely that artificial nutrition or human milk administration has altered α-tocopherol status. The sample size was relatively small, and it was a single-center setting. In addition, no body composition analysis was performed to distinguish fat and fat-free mass and their associations with α-tocopherol levels, and no longitudinal data were available.

## Conclusions

The present study demonstrated that about one-third of preterm neonates had very low α-tocopherol levels, while a simple anthropometric index, i.e., weight/length^2^ (g/cm^2^), related to α-tocopherol deficiency. The present study proposes that a simple measurement, routinely performed, can be used in a different direction to characterize nutrient status. Further studies, also conducted in developing countries, are needed to corroborate our results and prove the usefulness of the proposed index in the early detection of α-tocopherol deficiency and possibly other conditions. In this case, the clinical implications of the present and future findings include the identification of possibly α-tocopherol-deficient infants, which could prevent several infant pathologies, such as hemolytic anemia, intraventricular hemorrhage, bronchopulmonary dysplasia, ataxia, developmental delay, and others.

Thus, the identification of such "easy-to-measure" correlates of low vitamin E status, as proposed in the present study, is important to detect infants at high risk for α-tocopherol deficiency in clinical practice. Future studies with larger samples in several socioeconomic settings could use more sophisticated algorithms based on artificial intelligence and further guide clinical measurements with higher accuracy. Of course, awaiting further research evidence, the protocol of supplementation should be followed, as suggested by international bodies.
